# Some Accidents Observed on Horses of Pure English Stock

**Published:** 1896-07

**Authors:** F. Hendricks


Some Accidents Observed on Horses of Pure English Stock. The very considerable increase in the number of racing horses of pure English stock at Brussels and vicinity has given veterinarians many opportunities of observing various anomalies, the result of collisions or wounds, or happening during the regular running gait of the race. There was noted a series of lesions which may be classified as distention of the tendons, on which subject I hope soon to be able to publish a general work in collaboration with Prof. Degive. These lesions are most frequent in horses of pure English stock. They occur especially in the forelegs, either on the perforans or upon the perforatus, or on the suspensory ligament of the fetlocks. Each of these organs may be affected singly; in other cases only the perforans and the perforatus were found affected, while the suspensory ligaments of the fetlocks were normal. Twice the lesion on the perforans and the suspensory ligament was observed, the perforatus being intact. By distention of the tendons is understood a lesion which is called snapping. It consists essentially in a severance more or less complete of the constituent elements of the tendon. Such is at least the primary lesion. This distention is always accompanied by very rapid tearing of the vascular and nervous membranes. These phenomena are often marked; but in some cases the effusion of interstitial blood is slight and the increase in the size of the organ not noticeable. Usually there appears very rapidly a diffuse congestion, hot and sensitive to pressure, often extending along the whole length of the tendon from knee to fetlocks. In the beginning the pain is intense and the lameness marked; this painful sensation can be increased by pressure on the surrounding region. It is due not only to the rending of the nerve-extremities, but also to the compression exercised upon the same by the effusion of blood and by the inflammatory exudation which soon sets in; it is certain also that the irritating chemical action exercised upon the nervous elements by the products of inflammation explains to a certain degree the intense pain which often accompanies this accident. The leg which is the seat of the lesion is usually carried in advance, with the knee slightly bent and more or less rounded. In action the leg moves as a single piece, the flexion of the knee is very small, and the support is on the toe. It is not rare to see a marked febrile reaction appear at the same time. While the inflammatory phenomena disappear insensibly the effusion of blood is absorbed, but in the great number of cases it is observed that the interstitial exudation condenses and produces at the same time hypertrophy of the tendon; the pain disappears and the animal can walk and trot without showing the least lameness. More often, however, there remains a certain constraint in the gait, and the horse loses its great speed. There are some happy exceptions. A horse of pure English stock in which there was a complete lesion of the perforans, perforatus, and suspensory ligament was afterward excellent for the steeplechase. As to the prognosis, it depends upon the extent and the intensity of the lesion, and especially on the manner in which the horse has been handled after the accident.

*Treatment.* If the veterinarian is called at the beginning, it is especially important to combat the inflammation and relieve the pain. Two methods have given the best results, although they are diametrically opposed: hot water on the one hand and irrigation with cold water on the other. If recourse is had to the first method, a quilted bandage should be applied in the region of the metacarpal bone and kept constantly wet with water as hot as possible. The difficulties which are encountered in practice in applying this treatment led to the use of cold water. Where there is a water-supply, a stream of cold water can be conveyed to the metacarpal region by means of a rubber pipe. It is to be remarked that a very strong flow of water is not needed, the stream can be very small, provided that it be a *continuous* one. The good effects are increased by putting ice in the reservoir or barrel from which the water comes in order to make it as cold as possible.

If for any reason this treatment cannot be applied, it may be replaced advantageously by a quilted bandage around the region affected, kept continually wet with an astringent solution, such as water slightly alcoholized, or, better still, 2 per cent. of alum in water containing 5 per cent. of alcohol; tincture of arnica (10 per cent) also gives good results. If the pain is intense and the animal supports itself very little on the leg affected, it is important to support the opposite leg by putting a bandage on the fetlock and on the cannon bone. It is necessary to continue this mode of treatment for the first six or seven days; usually a marked amelioration is noticed at the end of this time. Cure is hastened by having a sort of massage treatment applied to the whole region for twenty minutes twice a day with a salve composed as follows: Mercurial ointment, 50 grams; extract of hemlock, 10 grams; iodide of potassium, 150 centigrams. The reporter has had markedly beneficial results with this treatment when the grooms have had the perseverance and the strength to carry out the massage in a suitable manner. When not certain that the massage will be carried out, I prefer to apply a blister, especially the ointment prescribed by Prof. Degive, and which is frequently used at the clinic in Cureghem: Vaseline (white), 160 grams; corrosive sublimate p., cantharides p., of each, 12 grams. Usually two applications are made at an interval of twelve hours.

If in spite of this treatment congestion still remains with a tendency to become chronic, recourse should be had to cauterization. The fire is applied in an ordinary way, and between the lines the fine points are put, piercing the skin as far as the subcutaneous cellular tissue. In certain cases the points are allowed to penetrate into the tendon. The results secured have sometimes been surprising; last year cauterization was applied in the manner described to a thoroughbred whose perforans and perforatus were both snapped. The result was so favorable that the horse lately won a brilliant victory at Auteuil, beating a number of excellent French horses. Great importance is attached to the manner in which the horse is used after treatment. In the reporter's opinion each horse should rest at least six months without doing anything; gentle exercise, massage, and cold-water applications being used.

*Fracture of the Scapulum.* Lately Mr. V. was racing a little thoroughbred mare of remarkable speed, which had carried off thirty-two prizes in 1895, of which twenty-four were firsts. Starting at the end of the group the mare found herself abruptly on three legs; the off foreleg would no longer give support. The jockey had heard at the same time a dry sound analogous to that produced by a club upon a box. Dismounting he discovered a very pronounced congestion of the olecranon muscles and of the region about the shoulder. The reporter saw the animal an hour later and found her suffering extreme pain; the eye fiery, the nostrils dilated, leaping from right to left in the air. She struggled so that he did not succeed in examining her thoroughly, fearing to be knocked down. The congestion pointed to a fracture, but he was compelled to put off his examination until the next day. The mare was supported in slings and applications of cold water on the shoulder ordered. The next morning to my great astonishment I found the mare dead, and it is probable that the animal had succumbed to the unendurable pain. At the autopsy a compound fracture of the shoulder was found. The scapula was literally reduced to bits, especially at the plane of the neck. At least a score of bony pieces was found in the solution of continuity. It is probable that the fracture was not so compound at the beginning; the violent exertion of the animal had caused small supplementary fractures. In this case there was no external wound; the accident happened while the animal was at full gallop.—F. HENDRICKS, in *Annales de Médecine Vètèrinaire*, Jan. and Feb. 1896.

